# Unexpected Role for *Helicobacter pylori* DNA Polymerase I As a Source of Genetic Variability

**DOI:** 10.1371/journal.pgen.1002152

**Published:** 2011-06-23

**Authors:** María-Victoria García-Ortíz, Stéphanie Marsin, Mercedes E. Arana, Didier Gasparutto, Raphaël Guérois, Thomas A. Kunkel, J. Pablo Radicella

**Affiliations:** 1 CEA, Institut de Radiobiologie Cellulaire et Moléculaire, UMR 217 CNRS/CEA, Fontenay aux Roses, France; 2 Laboratory of Molecular Genetics and Laboratory of Structural Biology, National Institute of Environmental Health Science, National Institutes of Health, Research Triangle Park, North Carolina, United States of America; 3 CEA, Institut Nanosciences et Cryogénie, Grenoble, France; 4 CEA, iBiTecS, Gif sur Yvette, France; 5 CNRS, URA 2096, Gif sur Yvette, France; Université Paris V, INSERM U571, France

## Abstract

*Helicobacter pylori*, a human pathogen infecting about half of the world population, is characterised by its large intraspecies variability. Its genome plasticity has been invoked as the basis for its high adaptation capacity. Consistent with its small genome, *H. pylori* possesses only two bona fide DNA polymerases, Pol I and the replicative Pol III, lacking homologues of translesion synthesis DNA polymerases. Bacterial DNA polymerases I are implicated both in normal DNA replication and in DNA repair. We report that *H. pylori* DNA Pol I 5′- 3′ exonuclease domain is essential for viability, probably through its involvement in DNA replication. We show here that, despite the fact that it also plays crucial roles in DNA repair, Pol I contributes to genomic instability. Indeed, strains defective in the DNA polymerase activity of the protein, although sensitive to genotoxic agents, display reduced mutation frequencies. Conversely, overexpression of Pol I leads to a hypermutator phenotype. Although the purified protein displays an intrinsic fidelity during replication of undamaged DNA, it lacks a proofreading activity, allowing it to efficiently elongate mismatched primers and perform mutagenic translesion synthesis. In agreement with this finding, we show that the spontaneous mutator phenotype of a strain deficient in the removal of oxidised pyrimidines from the genome is in part dependent on the presence of an active DNA Pol I. This study provides evidence for an unexpected role of DNA polymerase I in generating genomic plasticity.

## Author Summary

*Helicobacter pylori* is the main cause of ulcers and gastric cancers. One the characteristics of this bacterial species is that it displays an amazing capacity to change its genetic information. This genetic variability provides *H. pylori* with an adaptation potential that allows it to successfully colonise the stomach of about half the human population. Here we identified a surprising source of genomic plasticity in an enzyme also involved in the maintenance of DNA integrity. Indeed, we show that DNA polymerase I, one of the only two DNA polymerases that are found in *H. pylori*, although essential for DNA replication and repair, contributes to mutagenesis due to its biochemical characteristics.

## Introduction

Phenotypic selection from a pool of genetic variants present in their population allows prokaryotes to successfully adapt to specific niches and changing environments. The gram-negative bacterium *Helicobacter pylori* is one of the most successful human pathogens. Indeed, it colonises the stomach mucosa of about half the human population, triggering pathologies that span asymptomatic chronic gastritis, peptic ulcers and cancer [1]. The study of natural isolates suggests that the genetic diversity of *H. pylori* exceeds that recorded in all other bacterial species studied. Moreover, it is now clear that even in the course of infection of a single individual, *H. pylori* strains display high rates of allelic variation [2], [3]. This enhanced ability to change and the consequent advantage of counting upon a large pool of variants from which to select the most-fit combinations have been proposed to facilitate adaptation within a host as well as colonisation of new hosts [4], [5]. Therefore, besides its clinical importance, the amazing genetic variability of *H. pylori* makes it an excellent model for the analysis of the molecular mechanisms underlying microbial phenotype evolution.

At the origin of the allelic variability are nucleotide changes that can arise from replication errors either spontaneous or induced by DNA lesions. *H. pylori* displays a high rate of mutations, accounted not only by the lack of mismatch repair system [6], [7] but also by the exposure to genotoxic stresses during infection leading to the formation of DNA lesions [8], [9]. While replicative DNA polymerases are highly accurate and efficient in replicating undamaged DNA, the presence of abasic sites or modified bases will often impede the progression of the replication fork. It is now well established that in most organisms DNA polymerases exist that are capable of substituting for the replicative polymerase and facilitate translesion synthesis (TLS) allowing the replication machinery to overcome the blockage. In many cases, TLS is associated with the acquisition of heritable mutations induced by the incorporation by the TLS polymerase of an incorrect nucleotide opposite the lesion [10].

In agreement with the low functional redundancy in its DNA repair pathways, analysis of the *H. pylori* genome sequences predicts the presence of only two putative DNA polymerases. Indeed, six genes code for the replicative polymerase subunits, while one gene, *HP1470* in the reference strain 26695, codes for a putative DNA Polymerase I. *E. coli* Pol I was the first DNA polymerase discovered and is the most abundant one [11]. The Pol I bacterial DNA polymerases are multifunctional proteins. In most bacterial species Pol I presents two distinct functional domains, a 5′ - 3′ exonuclease N-terminal domain and a larger C-terminal domain (Klenow fragment) harbouring the polymerase and the associated proofreading 3′ – 5′ exonuclease catalytic sites [12]. The 5′-3′ exonuclease activity allows the removal of the RNA primers of the Okazaki fragments during DNA replication [13]. The gap-filling capacity of Pol I not only participates in the replication of the lagging strand but also in DNA excision repair and in recombination.

The absence of other predicted DNA polymerases in *H. pylori*, in particular of those capable of TLS, raises several questions regarding the distribution of roles between the two DNA polymerases during replication and repair. To investigate these issues we characterised the protein coded by *H. pylori polA* gene and showed that it is able to bypass blocking lesions. Based on our genetic results we conclude that *H. pylori* DNA polymerase I, albeit its important role in cell viability and DNA repair, contributes to mutagenesis during normal chromosome replication and therefore to the plasticity of the genome.

## Results

### *HP1470* codes for a bona fide DNA polymerase I devoid of proofreading activity

**Figure 1:**
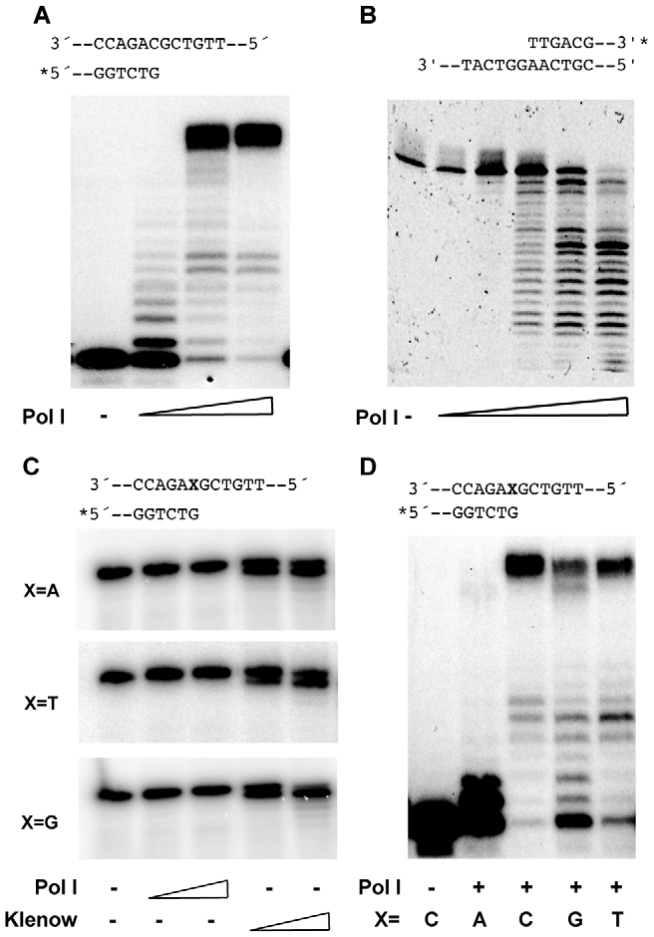
Pol I enzymatic activities. A. Primer extension. Increasing concentrations (0.1; 1 and 5 nM) of Pol I were used. B. 5′ – 3′ exonuclease. Concentrations of Pol I used were 10, 50, 100, 150 and 200 nM. C. 3′ – 5′ exonuclease on mismatched primers. Concentrations of *E. coli* Klenow fragment and Pol I were 25 and 100 nM. D. Mismatch extension. 5 nM Pol I was used. Substrates in A, C and D consisted in a 34-mer template oligonucleotide paired to 18-mer primers as described on top of each gel (Table S1). For B, the duplex was formed by a 62-mer (XV82) paired to a 31-mer (XV101) oligonucleotide (Table S1). In all cases representative gels of at least three independent experiments are shown.

The inferred absence of specialised TLS polymerases coded by the *H. pylori* genome [14], [15] prompted us to study the characteristics of the putative DNA polymerase I, one of the two predicted DNA polymerases of this pathogen. The protein sequence deduced for HP1470 suggested that the protein is a bona fide DNA Polymerase I orthologue. However, closer inspection of the amino acid sequence shows that even though the overall structure of the 3′ – 5′ exonuclease domain is likely to be preserved, at least three conserved residues - Asp^355^, Asp^424^ and Asp^501^ in *E. coli* DNA polymerase I - involved in metal binding and essential for the exonuclease catalytic activity [16]–[18] are missing (Figure S1). In order to verify the activities of the *H. pylori* DNA Polymerase I (herein Pol I), the protein was expressed in *E. coli* and purified to apparent homogeneity (Text S1 and Figure S2). As expected, Pol I displayed DNA–dependent polymerase (Figure 1A) and 5′ – 3′ exonuclease (Figure 1B) activities. In the polymerisation assays (Figure 1A), extensions beyond the expected full-length product could be detected. This has previously been shown to be characteristic of some DNA polymerases lacking a proofreading activity [19]. To verify the prediction of a lack of 3′- 5′ exonuclease activity, we then tested Pol I mismatch editing capacity. In conditions in which the *E. coli* Klenow fragment efficiently removed a mispaired base from the primer 3′-end, Pol I showed no exonuclease activity on substrates with different 3′-mismatches (Figure 1C). Moreover, with the possible exception of a G:A after which only one or two bases were incorporated, Pol I was able to extend primers with various mismatches at their 3′-end (Figure 1D).

### An essential role for the 5′ – 3′ exonuclease domain

In order to explore the role of Pol I *in vivo*, we generated *H. pylori* strains deficient in this protein. The constructs used to disrupt the gene were designed to replace the 2 kb central region of the gene with an antibiotic resistance cassette, leaving only 300 bp of the gene at each extremity. Interestingly, very few clones were obtained and in all cases analyzed, the cassette was inserted downstream of the expected site. Sequencing of five independent insertions showed that the first kilobase of the coding sequence was always preserved (Figure S3), potentially allowing the 5′ – 3′ exonuclease to be expressed. To rule out a sequence context bias for the insertion, we performed transformations with the same disruption cassette in a strain carrying an extra copy of the *polA* gene at the *ureA* locus. In this case the number of clones recovered was several orders of magnitude higher compared to those obtained from transformation of the wild type strain. Analysis of 22 independent insertions showed that 4 were in the ectopic gene and 18 in the *hp1470* locus. Interestingly, in all cases the insertion resulted in the expected product, a deletion starting 300 bp from the initiation codon, leading to the truncation of two thirds of the 5′ – 3′ exonuclease domain. Taken together, these observations strongly suggest that the 5′ – 3′ exonuclease activity coded by the N-terminal domain of Pol I is essential for viability.

### Pol I DNA polymerase activity is required for DNA repair but promotes mutagenesis

**Figure 2:**
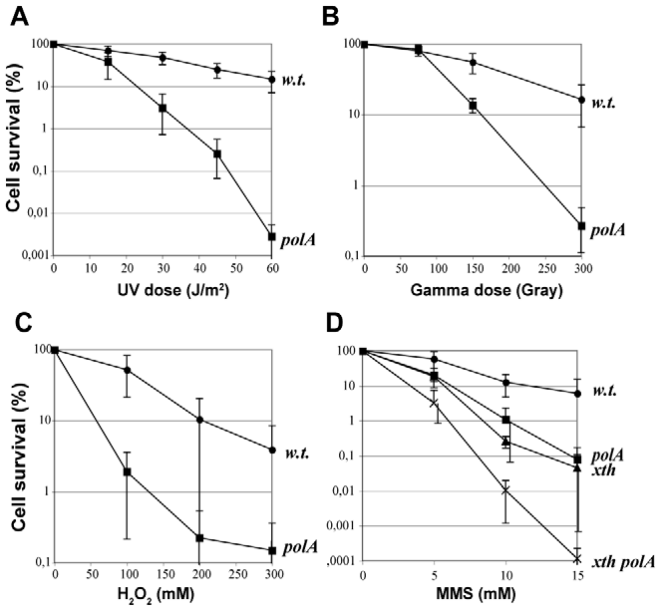
*polA* mutants are sensitive to genotoxic agents. Values correspond to the average of at least 4 independent determinations and their SD.

We then assessed the capacity of strains defective in DNA polymerase activity of Pol I (*polA*) to survive to various genotoxic treatments. The *polA* strains used correspond to those strains where the 3′ – 5′ exonuclease and polymerase domains are replaced by an antibiotic resistance cassette, leaving an intact 5′ – 3′ exonuclease domain, shown above to be essential. *polA* mutants are extremely sensitive to agents such as ionising radiation, UV light, hydrogen peroxide and the alkylating agent methyl-methanesulfonate (MMS) (Figure 2A–2D), all inducing different types of DNA damage. Interestingly, when *polA* was disrupted in a strain deficient in AP-endonuclease activity (*xth*) [20] there was an additive effect on the sensitivity to MMS, suggesting that Pol I participates in another pathway besides base excision repair. These results underscore the crucial role of Pol I in various DNA repair systems.

**Figure 3:**
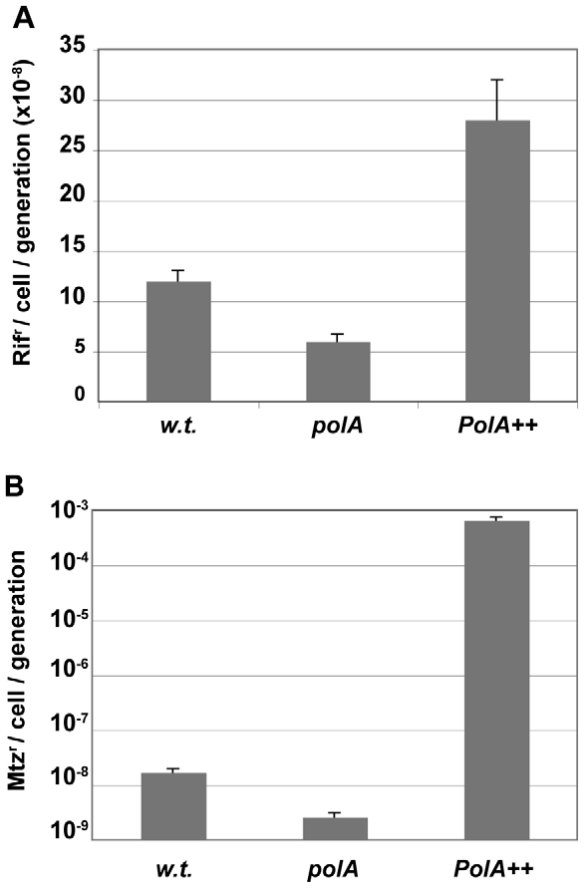
Pol I contributes to mutagenesis. A. Rates of spontaneous mutations to rifampicin resistance. B. Rates of spontaneous mutations to metronidazole (Mtz) resistance. Rates and SD were calculated by the method of the median from N independent cultures for wild type, *polA* and PolA++. N (rif) = 76, 50 and 11 and N (Mtz) = 20, 36 and 22 cultures, respectively.

To better characterise the *in vivo* functions of the protein, we tested the effect of the deficiency in Pol I on *H. pylori* spontaneous mutagenesis. The rate of base-pair substitutions was determined by monitoring the appearance of rifampicin-resistant (Rif^r^) colonies [9] (Figure 3A). Surprisingly, even though DNA polymerases I are involved in excision repair, inactivation of the polymerase activity of Pol I not only failed to increase base-pair mutation rates but resulted in a modest but significant hypo-mutator phenotype. Moreover, overexpression of Pol I driven by the strong *ureA* promoter resulted in a hyper-mutator phenotype thus indicating that Pol I *in vivo* generates base-substitutions.

Since the Rif^r^ mutagenesis test is limited to the detection of specific base substitutions, we used a forward mutation assay to explore a larger spectrum of genetic alterations. For such purpose, we determined the rate of mutations in the *rdx* gene, leading to resistance to metronidazole (Mtz^r^) [21]. The results showed a much more pronounced effect of Pol I on the Mtz^r^ mutation rates than in the case of Rif^r^. Indeed, inactivation of *polA* resulted in a 4-fold decrease in spontaneous mutations while its overexpression increased the rate of mutation by 500-fold (Figure 3B). Sequencing of Mtz^r^ isolates showed that the spectrum of mutations also changed. Indeed, sequencing of Mtz^r^ isolates showed that the 10-fold excess of mutants obtained in a wild type with respect to *polA* strain was due to 72- and 9-fold increases in the frequency of base substitutions and one base-pair frameshifts respectively, while the frequency of larger deletions or insertions was essentially unmodified (Table 1). The same trend was observed for the Pol I over-expressing strain where the increase in mutations was accounted for by the increase in base substitutions and one base pair frameshifts, with only 1 out of 36 clones analyzed displaying a change involving more than one base-pair (−2 deletion). Interestingly, in the overproducing strain the enhanced rate of base pair substitutions could essentially be accounted for by the increase in transversions. In conclusion, the excess mutations observed in strains expressing Pol I were essentially base substitutions or one nucleotide frameshifts. Taken together these data confirm that Pol I, although important for DNA repair, contributes to genetic variability mainly through the generation of single nucleotide polymorphisms.

**Table 1:** Sequence alterations in metronidazole-resistant mutants.

Alteration	Wild type		*polA*		PolA++	
	No. recovered*	Rate (10^−8^)	No. recovered*	Rate (10^−8^)	No. recovered*	Rate (10^−8^)
1 bp substitutions	19 (65)	43	6 (11)	0.6	14 (39)	10725
transversions	6 (21)	14	4 (7)	0.4	13 (36)	9960
transitions	13 (44)	29	2 (4)	0.2	1 (3)	321
1 bp insertion	2 (7)	4	16 (29)	1.6	15 (42)	11491
1 bp deletion	6 (21)	14	4 (7)	0.4	6 (17)	4596
>1 bp insertion	1 (3)	2	22 (41)	2.2	0 (0)	0
>1 bp deletion	1 (3)	2	6 (11)	0.6	1 (3)	550

*( ): percent of total events.

### Pol I has an accurate DNA polymerase activity

The results presented above prompted us to analyse whether, besides the lack of proofreading, Pol I harboured an intrinsic error-prone DNA polymerase activity. The fidelity of Pol I was determined during synthesis to fill a 407-nt single-stranded gap within a circular duplex M13mp2 DNA substrate. The gap contains the *lacZ* α-complementation sequence that serves as the target for detecting polymerisation errors that are detected as light blue and colourless plaques among blue plaques resulting from correct synthesis [22]. The DNA products of gap filling by Pol I yielded a *lacZ* mutant frequency of 0.15%. This frequency is lower than values obtained after gap filling by several other exonuclease-deficient family A polymerases, including 0.57% Klenow fragment polymerase [23], 0.75% for *Thermus aquaticus* polymerase [24], 1.6% for exonuclease-deficient T7 polymerase [23], [24], and 0.62% for exonuclease-deficient pol γ [25]. Thus *H. pylori* Pol I is among the most accurate exonuclease–deficient members of the family A polymerases when copying an undamaged DNA template *in vitro*.

### Translesion synthesis by Pol I

**Figure 4:**
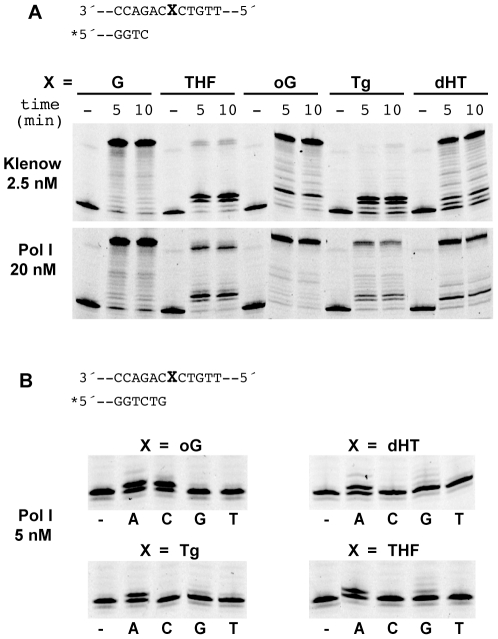
Translesion synthesis by Pol I. A. Translesion synthesis by *E. coli* Klenow fragment (2,5 nM) and Pol I (20 nM) on 34-mer templates harbouring a single lesion (X) at position 16. The polymerases have to add two nucleotides before encountering the lesion. oG = 8-oxoguanine; dHT = di-hydrothymidine; Tg = thymine-glycol and THF = tetra-hydrofurane, an abasic site analogue. The concentrations used for the two DNA polymerases were chosen for yielding the same activity on undamaged DNA. B. Nucleotide selection. Extension reactions directly on a lesion were carried out for 5 min in the presence of a single dNTP (0.1 mM) as indicated for each lane.

The apparent contradiction between the fidelity of the Pol I DNA polymerase activity on undamaged DNA and its role in the generation of mutations prompted us to further investigate the enzymatic characteristics of Pol I. The lack of proofreading activity, the consequent capacity to elongate from mismatches and the spectrum of mutations it generates are reminiscent of TLS polymerases. We directly addressed this possibility by determining the ability of purified Pol I to bypass DNA lesions present in the template strand. Among the lesions tested, some, like the abasic (AP) site analogue tetrahydrofurane (THF) and thymine glycol (Tg) are known to impose a blockage to normal DNA replication [26], [27] while others like 8-oxoguanine (8-oxoG) do not, but have a miscoding potential [28]. In the case of *E. coli* Klenow fragment, DNA synthesis is indeed strongly blocked opposite Tg and THF residues present in the template strand (estimated bypass efficiencies: 5 and 11% respectively). Conversely, albeit also partially blocked, *H. pylori* Pol I is able to synthesise through these lesions (Figure 4A) (estimated bypass efficiencies: 61 and 48%, for Tg and THF respectively).

We next examined the single nucleotide insertion profile promoted by Pol I opposite the various lesions. Consistently with the described coding capacity of 8-oxoG, Pol I introduces both A and C opposite the oxidised guanine (Figure 4B). For the other lesions tested DNA Pol I follows the A-rule, introducing preferentially adenine opposite the lesion. In the case of the abasic site analogue addition of G opposite THF is also observed (Figure 4B).

To confirm that the Pol I-dependent *in vivo* mutagenesis could be at least partly related to the presence of DNA lesions, we analysed the effect of Pol I on the spontaneous mutagenesis in strains lacking Nth, the DNA glycosylase responsible for the removal of oxidised pyrimidines from DNA. As previously reported [9], an *nth* mutant has a 4-fold higher mutation rate than its parental strain (Table 2). Inactivation of *polA* in an *nth* background resulted in partial reduction of the mutator phenotype induced by the lack of Nth, strongly suggesting that a fraction of unrepaired Nth substrate lesions are normally bypassed by the DNA Pol I. This result is consistent with a contribution of Pol I to mutagenesis through TLS.

**Table 2:** Rates (×10^−8^) of spontaneous mutation to Rif^r^.

Strain	Mutation rate ± SD
26695	12±1
*nth*	49±5
*polA*	6±0.7
*nth polA*	30±5

## Discussion

The success of *H. pylori* in colonising a large fraction of the human population has been attributed to its adaptation capacity based, in turn, on the genetic diversity of the species. At the basis of the remarkable genetic variation found in *H. pylori* are nucleotide polymorphisms that can be rapidly propagated by recombination between strains [29]. *In vivo* generation of allelic diversity is driven by mutation rates significantly higher than those of most other bacteria [6]. Several mechanisms have been suggested as contributing to the high mutation frequency of this pathogen, starting with the lack of homologues of many DNA repair genes known to be involved in maintaining the genetic stability in other bacterial species [4]. Among the most remarkable absences is probably that of a mismatch repair system [6], [7] capable of removing incorrect bases introduced during replication.

The work presented here unveiled another mechanism contributing to *H. pylori* high mutation rates. We showed that *H. pylori* strains deficient in DNA Pol I polymerase activity have reduced mutation rates indicating that DNA polymerase I actively participates in generating allelic diversity. This constitutes a surprising role for a protein associated with DNA repair and replication in all the studied bacterial models. The sensitivity of *polA* strains to various genotoxic agents confirms that *H. pylori* Pol I is involved in various DNA repair pathways such as recombination and base excision repair. However, their hypomutator phenotype is in contrast with the 7- to 10-fold-higher spontaneous mutation frequency in *E. coli* Pol I-deficient strains [30]. Our results, together with the absence of DNA polymerases other than DNA Pol I and Pol III, suggest that the *polA* gene in this species has been selected for coding a DNA Pol I capable of fulfilling extra functions allowing increased mutation rates. Indeed, we showed that DNA Pol I can not only extend mismatched primers, but also bypass DNA lesions that would normally block the replicative polymerase as well as DNA Pol I homologues from other bacteria. How can those characteristics contribute to increase mutagenesis?

Spontaneous or induced DNA damage is constantly generated in the genome [31]. In most organisms TLS polymerases allow replication across damaged DNA avoiding the blockage of the replication machinery. In *E. coli*, the SOS response includes the expression of TLS polymerases that allow survival in such situations at the expense of induced mutations. In spite of the lack of evidence for an SOS response system, it is now clear that *H. pylori* takes advantage of stress-induced DNA damage to mutate [8], [9]. Such a response necessitates a DNA polymerase capable of performing mutagenic TLS [10]. Unlike what was shown in *B. subtilis*, where Pol I, also lacking the proofreading function, acts in concert with TLS polymerases PolY1 and PolY2 to bypass lesions [32], the biochemical activities of *H. pylori* Pol I unveiled in this work would be sufficient to accomplish the task. The hypo-mutator phenotype of the *polA* strains and the lack of specialised TLS polymerases are consistent with this view. So is the increased proportion of transversions among base substitutions found in the Pol I overproducing strain with respect to the wild-type (13/14 versus 6/19), as expected for the bypass of non-coding lesions. The partial dependence on a functional DNA Pol I of the increased mutagenesis of an *nth* strain provides further support for this hypothesis. The oxidative stress generated by infection-induced inflammation, the acidic medium of the stomach together with the limited set of *H. pylori* DNA glycosylases involved in base excision repair [20], [33] favour the formation and persistence of stress-induced damage in DNA, including modified bases and abasic sites [9], [34]. Therefore, it is likely that beyond its functions in DNA repair, DNA Pol I plays an important role in the survival of the bacteria during infection by allowing the replication of damaged DNA and, concomitantly, by contributing to generate allelic diversity in response to the stress.

From the enzymatic point of view, *H. pylori* polymerase I combines two antagonistic properties not usually found within the same enzyme. Although it exhibits high accuracy on undamaged DNA, it is able to efficiently bypass several types of lesions and can extend mismatched primers. How can such a plasticity be understood in the context of a single enzyme? Regarding other Pol I homologues, accuracy was shown to be exquisitely controlled through a closing mechanism of the fingers domain involving a tight packing between the active site residues and the nucleotide to be inserted. Residues in the so-called O-helix were shown to actively disfavour misincorporation [35], [36]. Mutation Y766S within the O-helix of *E. coli* Klenow polymerase led to a more open active site and favoured lesion bypass at the expense of fidelity [37], [38]. Similarly, the more open active site of TLS polymerases, such as that of yeast Polη or the archaeal Dpo4, is a major determinant to account for their ability to accommodate bulky lesions [39], [40]. As a first hypothesis, we thought that *H. pylori* Pol I active site might have a special open structure particularly tolerant to mispairs insertions. However, mapping the conservation of the sequences of both Taq and H. pylori Pol I at the structure of Taq polymerase showed that both sequences are strictly conserved all along the active site groove (Figure S4). In particular, residues of the O-helix involved in the steric-gate mechanism are identical. Consequently, both the high accuracy of PolA on undamaged DNA and the conserved nature of the active site support that, unlike other TLS polymerases, *H. pylori* Pol I permissiveness is not due to a more open cavity. Consistently we have not been able to detect a significant mutagenesis induced by UV (data not shown). One might suppose that subtle dynamical properties of the enzyme allow accommodation of small lesions but not necessarily bulky lesions. A large body of evidence suggests that this is the case for other members of the Pol A family, including *E. coli* Klenow fragment. Indeed, these polymerases were shown to be able to incorporate a nucleotide opposite AP sites and products of cytosine or thymine oxidation (Tg, urea, uracyl-glycol and others) although not always to elongate from it [19], [26], [41]–[44]. Moreover, inactivation of the proofreading activity of Klenow allows the bypass of most of these lesions [19], [43], [45]–[47]. Interfestingly, two higher-eukaryote members of the family lacking a 3′-5′ exonuclease domain, DNA polymerases θ and ν, have been shown to be proficient for bypassing Tg and abasic sites [48]–[50]. Taking into account the strong structural conservation predicted for the active sites of *H. pylori* Pol I and the other members of the family (Figure S4), the work cited above supports the notion that the loss of proofreading activity can account for the capacity of Pol I to bypass AP sites and non-bulky damaged bases. In the case of AP sites this activity will contribute to mutagenesis either by incorporating in three out of four events the wrong nucleotide or by inducing frameshifts [47].

Further support for a role of TLS by Pol I in *H. pylori* mutagenesis, comes from the results showing that inactivation of Pol I partially complements the mutator phenotype of an *nth* strain (Table 2). *H. pylori* Nth is the only DNA glycosylase in this organism capable of removing oxidised pyrimidines from DNA [9]. Many of the products of thymine and cytosine oxidation are pre-mutagenic lesions. In particular, oxidised derivatives from cytosine as 5′-hydroxycytosine, uracyl-glycol and 5′-hydroxyuracyl [46], [51]–[54] but also from thymine [46], [51]–[54] have been shown to be bypassed by proofreading-deficient DNA polymerases and to be mutagenic [55], [56].

Besides TLS, another, non-exclusive, mechanism can be invoked for the role of *H. pylori* Pol I in mutagenesis. The essential character of the 5′ - 3′ exonuclease domain of *H. pylori* Pol I strongly suggest that this activity is required for Okazaki fragment processing, even in the absence of the other protein activities. Recently, elegant genetic experiments established that *E. coli* Pol I proofreading activity plays a crucial role in chromosomal replication fidelity [57]. The model put forward by the authors proposes that Pol I performs 1–2% of lagging strand synthesis. They show that inactivation of the 3′ - 5′ exonuclease activity leads to a mutator phenotype with a strong bias towards lagging strand mutations. The mutator phenotype observed in *polA* strains even in the absence of all three TLS polymerases is also consistent with a proposed role for *E. coli* Pol I proofreading activity during replication [58]. In the case of *H. pylori*, despite accurate polymerase activity of Pol I, its lack of proofreading capacity could contribute to mutagenesis during Okazaki fragment processing. In the absence of Pol I DNA polymerase activity, the replicative polymerase is the only candidate to perform lagging strand synthesis. Because of its high fidelity, lower mutation rates are expected. Conversely, over-expression of Pol I can lead to a more extensive processing of Okazaki fragments, therefore increasing the fraction of lagging strand synthesis performed by this enzyme and leading to a higher level of replication error rates.

In conclusion, independently of the relative contributions of Pol I to TLS and lagging strand synthesis, the results presented here strongly support the hypothesis by which in *H. pylori* the loss of proofreading activity of this DNA polymerase has been selected for increasing genome plasticity.

## Materials and Methods

### *H. pylori* strains and growth conditions

All *H. pylori* strains used were in the 26695 genetic background [15]. To generate gene-specific mutants, the corresponding open-reading frame (ORF) cloned into pILL570 was disrupted, leaving 5′ and 3′ ends (300 bp) of the gene, by a nonpolar kanamycin- (Km), apramycin- (Apr) or chloramphenicol (Cm) resistance cassette [59], [60]. To generate the Pol I over-expressing strain, the *HP1470* ORF was inserted into pADC vector, downstream of the *ureA* promoter, as described [61]. Plasmids were introduced into *H. pylori* strains by natural transformation and recombinants were selected after 3 to 5 days of growth on either 20 µg/ml Km, 12.5 µg/ml Apr or 8 µg/ml Cm. Allelic replacement was verified by PCR. As described in the Results section, the *polA* mutants used correspond, unless specified, to the replacement of the equivalent of the Klenow fragment by the resistance cassette, leaving the 5′ to 3′ exonuclease domain intact. Double mutants were obtained by plasmid or genomic DNA transformation of single mutant or by mixing two mutant strains together before plating the mix on double selection. *H. pylori* cultures were grown at 37°C under a microaerobic atmosphere on BAB, blood agar base medium supplemented with an antibiotic mix and 10% defibrillated horse blood.

### Sensitivity and mutagenesis assays

For all experiments, *H. pylori* strains were initially grown for 24 hr on plates with BAB medium. UV, MMS and gamma irradiation sensitivity assays were performed as described [33], [62]. For chemical oxidative stress treatment, *H pylori* (OD_600_ = 1) cell suspensions were incubated with different concentrations of hydrogen peroxide (100, 200 and 300 mM). Cells were washed 10 min later, diluted with peptone broth and plated on BAB plates. Survival was determined as the number of cells forming colonies on plates after a given treatment divided by the number of colonies from non-treated cells. Assays to determine spontaneous mutation rates were performed as described [7].

### Activity assays

All assays were performed at 37°C for 30 min in 20 µl reactions containing 10 mM Tris-HCl (pH 7.9), 50 mM NaCl, 10 mM MgCl_2_, 1 mM Dithiothreitol, 2.5 nM DNA substrate (see Text S1 and Table S1) and variable concentrations of Pol I (specified in the figure legends). 0.1 mM of either all four dNTPs or each dNTP individually was included in the reactions except for the exonuclease activity assays. When required as a control, Klenow fragment DNA polymerase (Roche) was used. Reactions were stopped by adding loading buffer (10 mM EDTA, 95% (v/v) formamide, 0.03% (w/v) bromophenol blue, 0.03% (w/v) xylene cyanol) and subjected to electrophoresis in 8 M urea-containing 20% polyacrylamide gels. Gels were visualised and quantified using a Molecular Dynamics PhosphorImager. According to Koskoska et al. [63], bypass probabilities were calculated as the proportion of DNA synthesis products extended beyond the lesion. Bypass efficiencies were then calculated for each enzyme and each substrate as the ratio of the bypass probability of a specific damaged base with respect to that of an undamaged nucleotide in the same position.

### M13mp2 fidelity assay

The assay was performed as described previously [22]. Gap-filling DNA synthesis was performed in a reaction mixture (25 µl) containing 50 mM Tris (pH 6.8), 50 mM NaCl, 10 mM MgCl_2_, 1 mM DTT, 0.2 mM each of dNTP and 0.2 nM of gapped M13mp2 DNA substrate. Reactions were initiated by adding Pol I, incubated at 37°C for 30 min, and terminated by adding EDTA to 20 mM. When DNA products were analyzed by agarose gel electrophoresis [22], the majority of the gapped molecules were filled to completion. However, a minority of DNA products migrated as if synthesis had paused at the palindrome just upstream of the open reading frame of the *LacZ* gene. In this minority population, only about 75% of the template used to score errors had been copied. As a consequence, the *lacZ* mutant frequency observed for the ensemble reaction products may slightly underestimate the error rate of *H. pylori* Pol I.

## Supporting Information

Figure S1 Multiple sequence alignment of polymerase I (*Escherichia coli*) and of polymerase A (*Helicobacter pylori*) homologs from various bacterial species spanning the region of the 3′-5′ exonuclease domain. The four positions involved in chelating the divalent metals (as shown in Figure 1) are highlighted by red triangles on top of the alignment. The aligned species were selected to sample species with or without the consensus site required for metal-binding in the 3′-5′ exonucleolytic site (name written in black or red, respectively). In every clade of the bacterial classification, polymerases lacking these residues can be found. Among ε proteobacteria, *Helicobacter hepaticus* is the closest species to *Helicobacter pylori* with an *a priori* functional 3′-5′ exonucleolytic site. Among γ proteobacteria, some species among the *Pseudomonas* clade do not contain the consensus site. Among the species tested, all β proteobacteria were found with a conserved functional site. Conversely, among the Firmicutes or Actinobacteria tested, none of them have the correct consensus site.
(TIF)

Figure S2 Pol I protein. *H. pylori* Pol I overexpressed in *E. coli* was purified to near homogeneity as judged by analysis on 10% SDS polyacrylamide gel electrophoresis and staining with Coomassie blue.
(TIF)

Figure S3 Disruption of the *polA* gene. Wild type strains were transformed with the plasmid carrying the *polA* ORF in which its central part was replaced by an antibiotic-resistance cassette (ATB^R^), leaving 300 base pairs (bp) of the ORF at each end. Sequence analyses indicated that all the antibiotic-resistant recombinants recovered had the ATB^R^ cassette inserted 998 bp downstream the initiation codon. The integration was likely to have occurred through a recombination between 7 bp repeats. (A) Expected recombination event. (B) Actual event in which the final product would allow the expression of the first 330 amino acids of the protein where resides the 5′ – 3′ exonuclease activity.
(TIF)

Figure S4 (A) Ribbon representation of a structure of the Taq polymerase (3LWM) with its various domains highlighted in yellow (3′-5′ exonuclease), red (palm), green (thumb) and cyan (fingers). DNA complexed to the polymerase is colored in purple (B) Surface representation of the Taq polymerase domain in the same orientation as in panel A. The surface is coloured with respect to the conservation between the sequences of the Taq and that of H. pylori PolA. Red color indicates the invariant positions while colors ranging from orange to pale yellow report residues with decreasing similarity. (C) A section of the surface representation highlights the O-helix residues engulfing the nucleotides as identical between both Taq and H. pylori PolA polymerase.
(TIF)

Text S1 DNA substrates preparation and Pol I expression and purification methods.
(DOC)

Table S1 Sequence of DNA oligonucleotides used for preparing substrates.
(DOC)
