# Supplementary figures and images for: Unexpected Role for Helicobacter pylori DNA Polymerase I As a Source of Genetic Variability
Source: PLoS Genet. 2011 Jun 23;7(6):e1002152. doi: 10.1371/journal.pgen.1002152 (PMC3121766; doi:10.1371/journal.pgen.1002152)

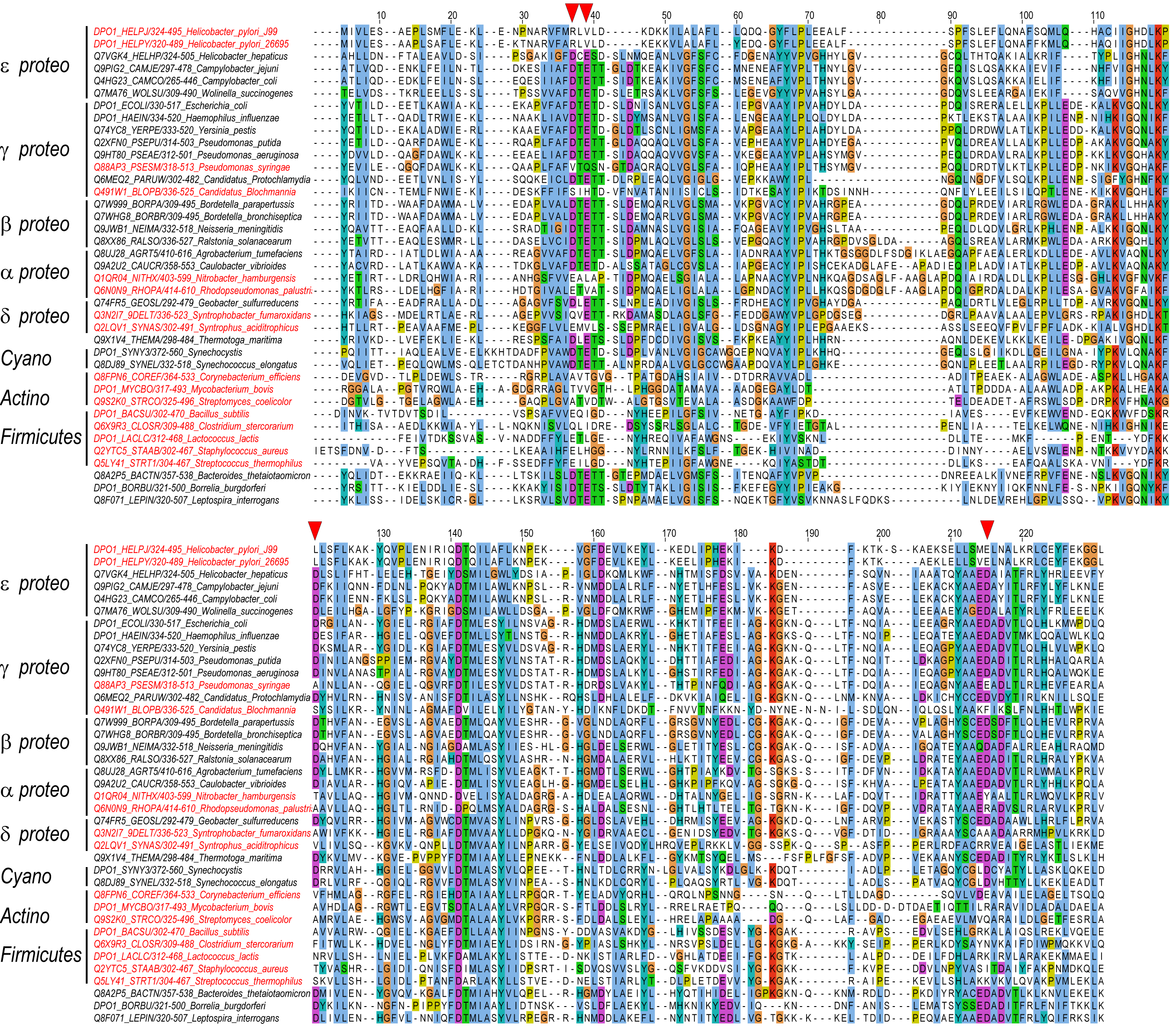

Supplement: Figure S1 — Multiple sequence alignment of polymerase I (Escherichia coli) and of polymerase A (Helicobacter pylori) homologs from various bacterial species spanning the region of the 3′-5′ exonuclease domain. The four positions involved in chelating the divalent metals (as shown in Figure 1) are highlighted by red triangles on top of the alignment. The aligned species were selected to sample species with or without the consensus site required for metal-binding in the 3′-5′ exonucleolytic site (name written in black or red, respectively). In every clade of the bacterial classification, polymerases lacking these residues can be found. Among ε proteobacteria, Helicobacter hepaticus is the closest species to Helicobacter pylori with an a priori functional 3′-5′ exonucleolytic site. Among γ proteobacteria, some species among the Pseudomonas clade do not contain the consensus site. Among the species tested, all β proteobacteria were found with a conserved functional site. Conversely, among the Firmicutes or Actinobacteria tested, none of them have the correct consensus site. (TIF) [file pgen.1002152.s001.tif]

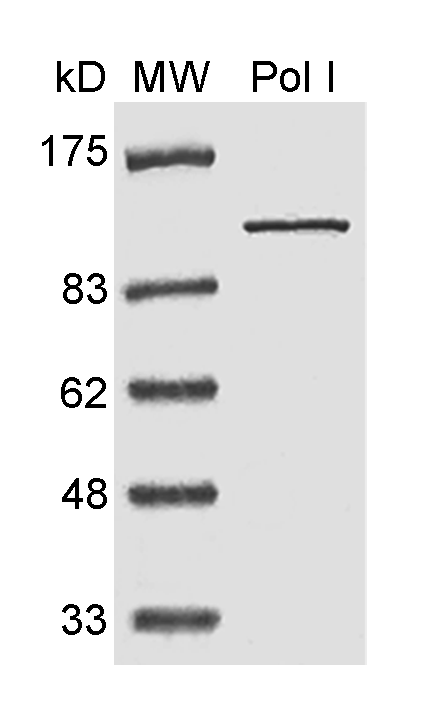

Supplement: Figure S2 — Pol I protein. H. pylori Pol I overexpressed in E. coli was purified to near homogeneity as judged by analysis on 10% SDS polyacrylamide gel electrophoresis and staining with Coomassie blue. (TIF) [file pgen.1002152.s002.tif]

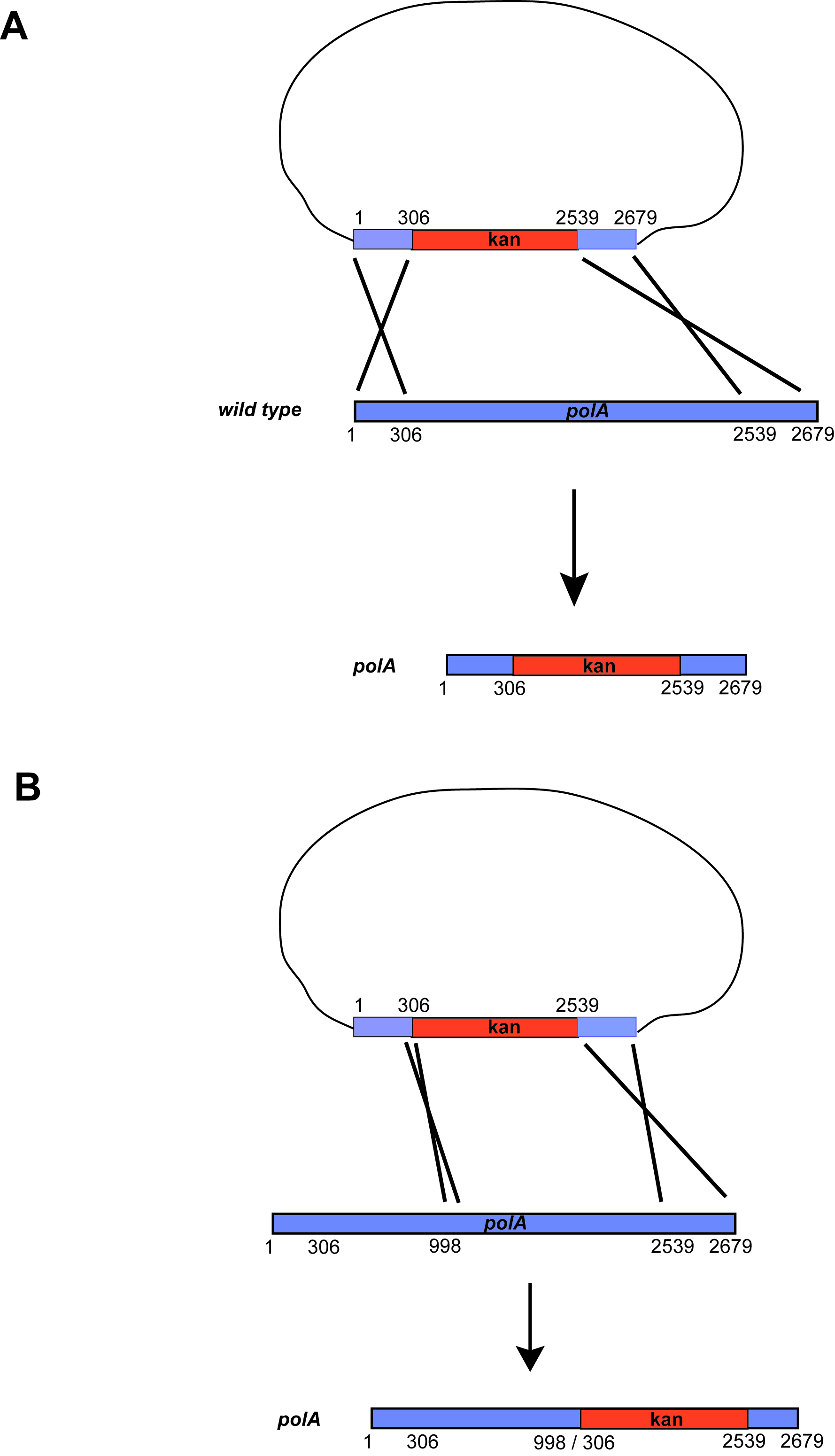

Supplement: Figure S3 — Disruption of the polA gene. Wild type strains were transformed with the plasmid carrying the polA ORF in which its central part was replaced by an antibiotic-resistance cassette (ATBR), leaving 300 base pairs (bp) of the ORF at each end. Sequence analyses indicated that all the antibiotic-resistant recombinants recovered had the ATBR cassette inserted 998 bp downstream the initiation codon. The integration was likely to have occurred through a recombination between 7 bp repeats. (A) Expected recombination event. (B) Actual event in which the final product would allow the expression of the first 330 amino acids of the protein where resides the 5′ – 3′ exonuclease activity. (TIF) [file pgen.1002152.s003.tif]

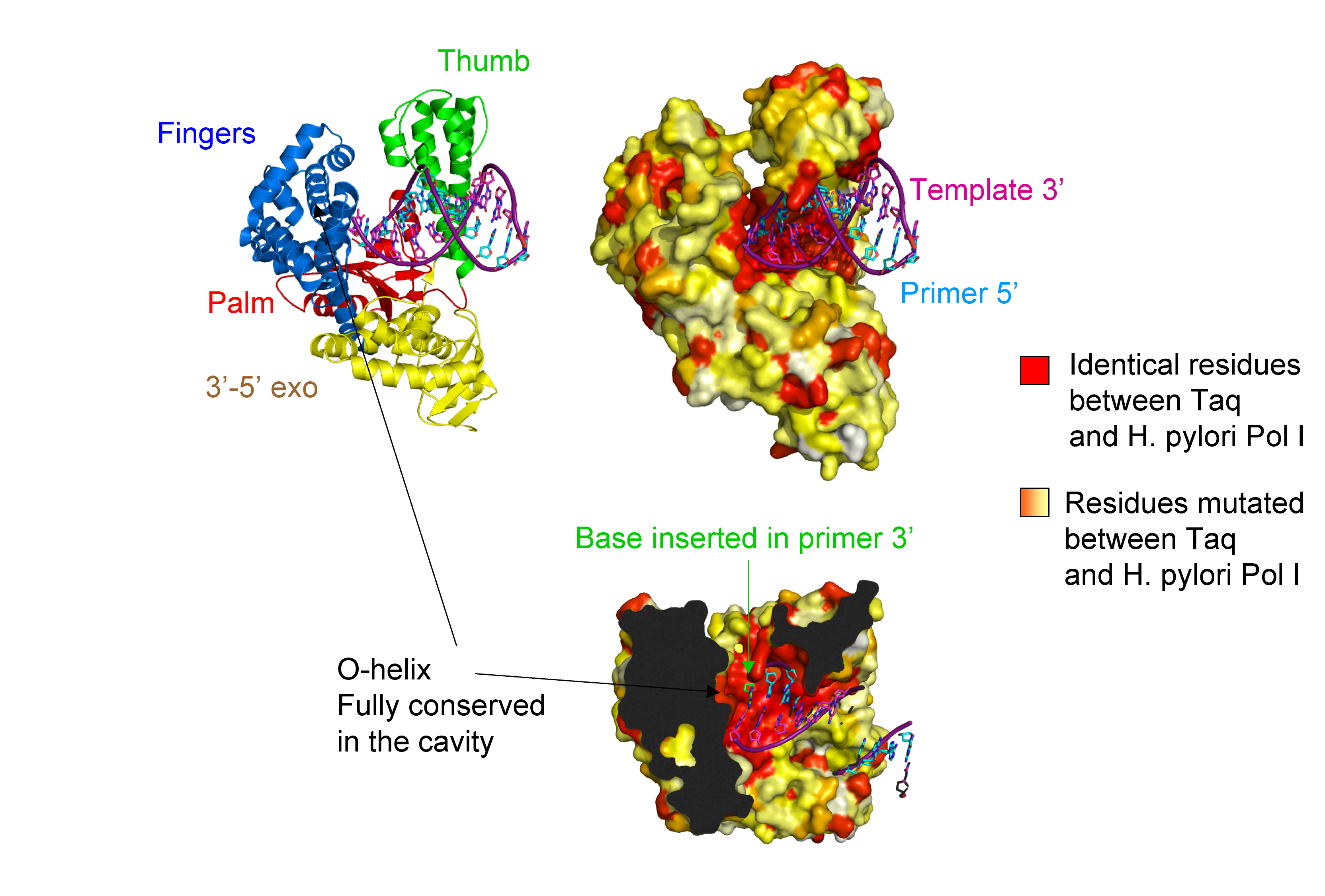

Supplement: Figure S4 — (A) Ribbon representation of a structure of the Taq polymerase (3LWM) with its various domains highlighted in yellow (3′-5′ exonuclease), red (palm), green (thumb) and cyan (fingers). DNA complexed to the polymerase is colored in purple (B) Surface representation of the Taq polymerase domain in the same orientation as in panel A. The surface is coloured with respect to the conservation between the sequences of the Taq and that of H. pylori PolA. Red color indicates the invariant positions while colors ranging from orange to pale yellow report residues with decreasing similarity. (C) A section of the surface representation highlights the O-helix residues engulfing the nucleotides as identical between both Taq and H. pylori PolA polymerase. (TIF) [file pgen.1002152.s004.tif]
