# Supplementary material for: Unexpected Role for Helicobacter pylori DNA Polymerase I As a Source of Genetic Variability
Source: PLoS Genet. 2011 Jun 23;7(6):e1002152. doi: 10.1371/journal.pgen.1002152 (PMC3121766; doi:10.1371/journal.pgen.1002152)
Supplement: Text S1 — DNA substrates preparation and Pol I expression and purification methods. (DOC) [file pgen.1002152.s005.doc]

**Supplementary Methods**

**DNA substrates.** With the exception of those carrying a thymine glycol, synthetic DNA oligonucleotides were obtained from Eurogentec. Tg oligonucleotides were synthesized as described (1). PAGE-purified oligonucleotides were labeled either at their 5’-ends using T4 polynucleotide kinase (New England Biolabs) and [γ-^32^P]ATP (3000 Ci/mmol; Amersham). Labeled primers were then hybridized with the template oligonucleotides (ratio 1:3) in the presence of 50 mM Tris-HCl (pH 7.5), 0.3 M NaCl, and heating to 90ºC for 10 min, before cooling to room temperature overnight. Oligonucleotide sequences used are listed in Table S1. For the 5’-3’ exonuclease activity determination the 31-mer was labeled at its 3’-end with deoxyadenosine [α-^32^P] cordycepine 5’ TP and a terminal transferase (New England Biolabs). This labeled oligonucleotide was hybridized as described above with a complementary 62-mer. The template-primer duplex used for monitoring DNA polymerase activity was the 34-mer template Compl-A annealed to the 16-mer primer 16-T. DNA Substrates used for mismatch extension and 3’-5’ exonuclease activity reactions were 34-mer Parental G-A, Parental G-T and Parental G-G as templates, hybridized with 18-mer 18-G as primer, giving rise to G-A, G-T and G-G substrates. Finally, for lesion bypass synthesis and single nucleotide extension reactions, 34-oxoG, 34-Tg and 34-THF were used as DNA templates, annealed with 18-G primer.

**Pol I expression and purification.**To produce Pol I, *E. coli* strain BL21 (DE3) harbouring pETM11-PolI was grown in 500 ml at 30 °C until OD reached 0.5. Expression was induced with 0.4mM of isopropyl-1-thio-D-galactopyranoside (IPTG) allowed to proceed at 20°C overnight. Cells were harvested, resuspended in 80 ml of lysis buffer (20 mM Phosphate, 500 mM NaCl, 2.5  % glycerol, 0,01 % triton X100, 0.4 mM AEBSF, 0.1 mg/ml lysosyme, pH 7.6) and lysed by sonication. The lysate was centrifuged at 4°C for 20 min at 100000 g. A first precipitation with 40 % saturation ammonium sulphate (AS) was performed. Pol I remaining was then precipitated from the resulting supernatant with 40 % saturation AS. The precipitated proteins were resuspended in 10 ml of A1000 buffer (20 mM Phosphate, 1 M NaCl, 5 % glycerol, pH 7.6) containing 5 mM Imidazol, and loaded on a 1.5 ml column of Nickel resin (Qiagen). The column was washed first with A1000 buffer containing 40 mM of Imidazole, then with A50 buffer (20 mM Phosphate, 50 mM NaCl 5 % glycerol, pH 7.6). The protein was eluted with same buffer with 500 mM Imidazole and loaded on a 1 ml heparin column (equilibrated in A50 buffer, eluted with a linear NaCl gradient in buffer A50 and collected at 500 mM NaCl. The samples were desalted in A50 buffer on PD10 columns (Amersham Biosciences) and loaded on a source S column (Amersham Biosciences). Pol I was then eluted with a linear NaCl gradient in buffer A50 and collected at 250 mM NaCl. The protein was stored at -20°C in the presence of 50 % of glycerol. They kept activities for at least 6 month.

1. Gasparutto D, Cognet S, Roussel S, Cadet J (2005) Synthesis of a convenient thymidine glycol phosphoramidite monomer and its site-specific incorporation into DNA fragments*. Nucleosides Nucleotides Nucleic Aci*ds 24:1831–1842.
