# Supplementary material for: Unexpected Role for Helicobacter pylori DNA Polymerase I As a Source of Genetic Variability
Source: PLoS Genet. 2011 Jun 23;7(6):e1002152. doi: 10.1371/journal.pgen.1002152 (PMC3121766; doi:10.1371/journal.pgen.1002152)
Supplement: Table S1 — Sequence of DNA oligonucleotides used for preparing substrates. (DOC) [file pgen.1002152.s006.doc]

**Table S1.** Sequence of DNA oligonucleotides used for preparing substrates

| Designation | Nucleotide sequence |
| --- | --- |
| Compl A | 5’-CGGTATCCACCAGGTCTGAGACAACGATGAAGCC-3’ |
| 16-T | (*)5’-GGCTTCATCGTTGTCT-3’ |
| Parental G-A | 5’-GGCTTCATCGTTGTCG**A**AGACCTGGTGGATACCG-3’ |
| Parental G-T | 5’-GGCTTCATCGTTGTCG**T**AGACCTGGTGGATACCG-3’ |
| Parental G-G | 5’-GGCTTCATCGTTGTCG**G**AGACCTGGTGGATACCG-3’ |
| 34-oxoG | 5’-GGCTTCATCGTTGTC**8oG**CAGACCTGGTGGATACCG-3’ |
| 34-Tg | 5’-GGCTTCATCGTTGTC**Tg**CAGACCTGGTGGATACCG-3’ |
| 34-THF | 5’-GGCTTCATCGTTGTC**THF**CAGACCTGGTGGATACCG-3’ |
| 18-G | (*)5’-CGGTATCCACCAGGTCTG-3’ |
| XV101 | TTG AGG CTG TCT AGA GGA TCC GAC TAT CGA T |
| XV82 | ATC GAT AGT CGG ATC CTC TAG ACA GCG TCA AGG TCA TTG GCA CTG GTA GAA TTC GGC AGC GT |

oG = 8-oxoguanine; Tg = thymine glycol; THF = tetrahydrofurane; (*) ^32^P label
